# Characterization of Three Distinct Loss-of-Function Cav2.3 Variants

**DOI:** 10.3390/ijms27094103

**Published:** 2026-05-03

**Authors:** Ivana A. Souza, Eder Gambeta, Mehdi Benkirane, Gerald W. Zamponi, Maria A. Gandini

**Affiliations:** 1 Department of Clinical Neurosciences, Cumming School of Medicine, University of Calgary, Calgary, AB T2N 4N1, Canada; iassisso@ucalgary.ca (I.A.S.); eder.deandrade@ucalgary.ca (E.G.); 2 Hotchkiss Brain Institute, Cumming School of Medicine, University of Calgary, Calgary, AB T2N 4N1, Canada; 3 Alberta Children’s Hospital Research Institute, Cumming School of Medicine, University of Calgary, Calgary, AB T2N 4N1, Canada; 4 Faculté de Médecine Montpellier-Nîmes, Université de Montpellier, 34000 Montpellier, Occitanie, France; m-benkirane@chu-montpellier.fr

**Keywords:** channelopathies, high voltage-activated Ca^2+^ channels, global developmental delay, autism spectrum disorder, epileptic encephalopathy, calcium channel

## Abstract

De novo variants in *CACNA1E*, the gene encoding the Cav2.3 voltage-gated calcium channel, are often associated with severe neurodevelopmental disorders, including developmental and epileptic encephalopathy. All reported variants up to date have exhibited gain-of-function effects on their biophysical properties. Here, we functionally characterize three pathogenic *CACNA1E* variants: H151L, M163T, and R1182C, using electrophysiology and structural modeling. M163T and R1182C exhibit depolarizing shifts in the voltage-dependence of activation, whereas R1182C also shows a reduced peak current density. H151L selectively slows recovery from inactivation. Our findings provide the first mechanistic evidence linking loss-of-function Cav2.3 pathogenic variants to variable neurological phenotypes, expanding the clinical spectrum of *CACNA1E* channelopathies.

## 1. Introduction

**Figure 1:**
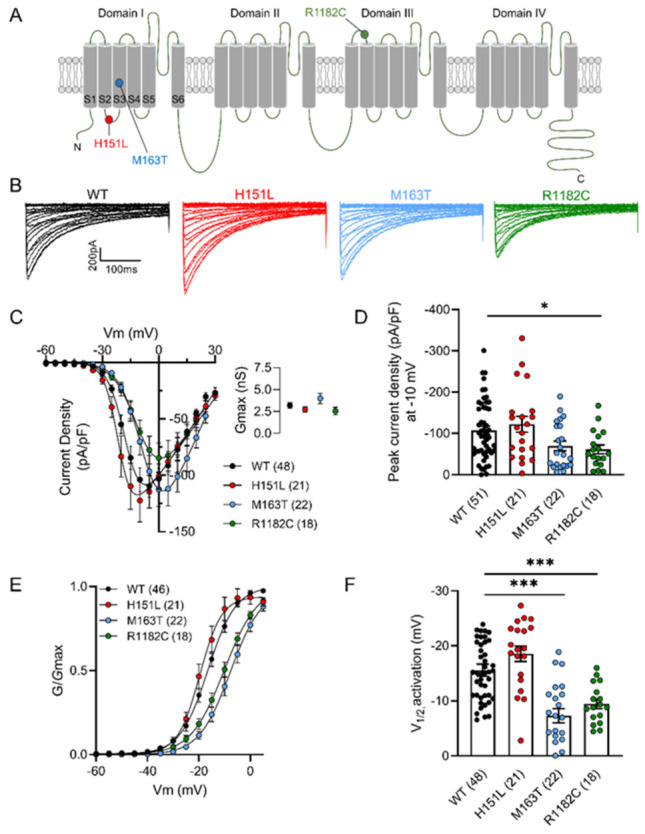
M163T and R1182C present a depolarizing shift in their activation curves. (**A**) Cav2.3 topology. Schematic representation showing the position of H151L, M163T, and R1182C pathogenic variants on the secondary structure of the Cav2.3 α1 subunit. The H151L variant is found in the intracellular loop between S2 and S3 segments of domain I. M163T resides within the S3 transmembrane segment of the same domain. Finally, R1182C is located in the extracellular connecting loop between S1 and S2 segments of domain III. (**B**) Representative whole-cell Ca^2+^ current traces recorded in response to depolarizing steps from −60 to 30 mV from a holding potential of −100 mV. (**C**) Average current density relationships for cells expressing Cav2.3 WT channels, and H151L, M163T, and R1182C pathogenic variants. Inset: Gmax values calculated using the modified Boltzmann equation for Cav2.3 WT channels, H151L, M163T, and R1182C pathogenic variants. (**D**) Average peak current density measure at −10 mV for Cav2.3 WT channels, and H151L, M163T, or R1182C variants. (**E**) Voltage-dependence of activation for cells expressing Cav2.3 WT channels, H151L, M163T, and R1182C pathogenic variants. (**F**) Mean half-activation potential values for cells expressing Cav2.3 WT channels, and H151L, M163T, or R1182C pathogenic variants. The numbers in the parentheses represent the number of cells recorded. Asterisks denote significance * 0.05, *** 0.001 levels.

High voltage-activated Ca^2+^ (Ca_V_) channels are membrane proteins comprising the pore-forming Ca_V_α_1_ and ancillary Ca_V_α2δ and Ca_V_β subunits [1]. Ca_V_α_1_ has 24 α-helical transmembrane segments divided into 4 domains (I–IV), each one with six segments (S1–S6; Figure 1A). The S1–S4 segments of each domain form the voltage sensor, whereas S5 and S6, and their connecting loop, form the ion-conducting pathway of the channel [2].

Among these, Ca_V_2.3 channels contribute to activity-dependent Ca^2+^ accumulation and selectively facilitate short-term plasticity and long-term potentiation [3], while their participation in neurotransmitter release evoked by single action potentials is low [4,5,6,7]. Moreover, they play a central role in shaping neuronal excitability and firing dynamics through functional coupling with different potassium channel subtypes, including SK, BK, and Kv4.2 channels [8,9,10,11], thereby regulating action potential repolarization, afterhyperpolarization, dendritic excitability, and interspike interval timing [10,11,12].

Pathogenic variants in the *CACNA1E* gene, which encodes the Ca_V_2.3α_1_ subunit, are associated with a severe early-onset developmental and epileptic encephalopathy (DEE). Affected individuals typically present neonatal or early infantile seizure onset, profound global developmental delay (GDD), severe axial hypotonia, spastic quadriplegia, congenital joint contractures, and macrocephaly [13,14,15]. Moreover, hyperkinetic movement abnormalities are frequently among the earliest clinical features [13,14]. Although epilepsy develops in the majority of them, a subset exhibits neurodevelopmental impairments such as developmental regression, autism spectrum disorder, and intellectual disability in the absence of overt seizures [13,15,16].

There is phenotypic variability observed among individuals carrying identical variants [15]. Moreover despite the broad expression of Cav2.3 channels in the brain, peripheral nervous system, cardiac myocytes, sperm, kidneys, pancreas, and gastrointestinal tractclinical manifestations predominantly affect neurological and musculoskeletal systems. This may potentially reflect age-dependent and tissue-specific expression patterns of distinct Cav2.3 isoforms [17,18,19,20,21,22].

Here, we describe the functional effects of three de novo missense mutations: p.H151L, p.M163T, and p.R1182C on Cav2.3 channel activity (Figure 1A). We report varying biophysical effects of these variants via electrophysiological characterization in tsA-201 cells. Our data reveal surprising loss-of-function effects rather than the typical gain-of-function associated with this channel.

## 2. Results

### 2.1. Clinical Characteristics

The three variants analyzed in this study are de novo missense variants in *CACNA1E* identified in symptomatic individuals. The H151L variant was reported in a 48-year-old female presenting with upper and lower limb spasticity, urinary incontinence, and progressive cerebellar ataxia. Brain MRI revealed marked cerebellar atrophy together with posterior supratentorial cortical atrophy. The patient’s symptoms onset occurred during childhood and had no history of epilepsy.

The M163T variant was identified in a 6-year-old male (Patient 1 reported by Royer-Bertrand et al. [15]), who presented with global developmental delay and developmental regression, including loss of communication skills and bladder control. He was also diagnosed with autism spectrum disorder and had no reported history of seizures.

The R1182C variant has been reported in ClinVar (Variation ID: 1251961). Among the submitted clinical reports, one individual was described with hydrocephalus, photophobia, dry skin, broad toes, and finger clinodactyly. This patient presented with seizures.

Although the three individuals present with partially distinct clinical phenotypes, all exhibit neurological involvement affecting motor and/or cerebellar systems. This shared clinical context, together with the presence of de novo *CACNA1E* variants, provides the rationale for comparative electrophysiological analysis of the three mutations.

### 2.2. R1182C Channels Have a Decrease in Peak Current Density

To assess the functional impact of the pathogenic Cav2.3 missense mutations H151L, M163T, and R1182C, each variant was introduced into the human Cav2.3 channel, and whole-cell Ca^2+^ currents were recorded from transiently transfected cells expressing Cav2.3 (WT or mutant) together with Cavβ1 and Cavα2δ-1 using the whole-cell patch-clamp technique. Representative current traces are shown in Figure 1B.

Figure 1C shows the average current density–voltage relationships (peak current amplitude normalized to membrane capacitance, Cm) obtained using 400 ms depolarizing steps from a holding potential of −100 mV. Compared to Cav2.3 WT, the M163T and R1182C variants exhibited current density–voltage relationships shifted toward more depolarized potentials, whereas H151L showed no significant shift. No significant differences in maximal conductance (Gmax) were observed (Figure 1C, inset).

Analysis of peak current density revealed a significant reduction in R1182C currents, indicating a loss-of-function (LoF) phenotype (Figure 1D).

### 2.3. M163T and R1182C Variants Alter Cav2.3 Gating Properties

To further characterize how these mutations affected channel gating, we analyzed voltage-dependent activation by building conductance-voltage (G-V) relationships (Figure 1E). The slope factor was significantly increased for both M163T (−4.99 ± 0.28, *p* < 0.002, one-way ANOVA) and R1182C (−5.84 ± 0.28, *p* < 0.0001) compared to Cav2.3 WT (−3.93 ± 0.17), suggesting an altered sensitivity to voltage for M163T and R1182C mutants.

In addition, the M163T and R1182C variants showed a depolarizing shift in the half-activation voltage (V½) relative to WT (Figure 1F). Specifically, V½ values were −15.6 ± 1.1 mV for WT, −7.3 ± 1.3 mV for M163T, and −9.5 ± 0.9 mV for R1182C, whereas H151L showed no significant difference (−18.6 ± 1.4 mV), indicating a LoF for M163T and R1182C variants.

### 2.4. Steady-State Inactivation Is Not Modified in Pathogenic Variants

**Figure 2:**
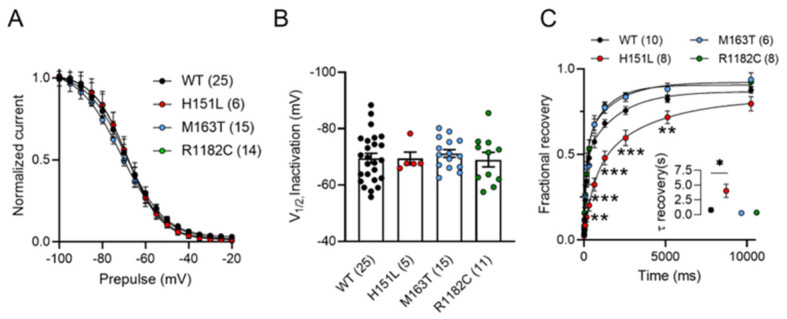
H151L presents a slowed recovery from inactivation. (**A**) Voltage-dependent steady state inactivation for cells expressing Cav2.3 WT channels, and H151L, M163T, or R1182C pathogenic variants. (**B**) Mean half-inactivation potential values for Cav2.3 WT channels, and H151L, M163T, and R1182C variants. (**C**) Fractional recovery from inactivation for Cav2.3 WT channels, H151L, M163T, and R1182C mutants. The numbers in parentheses represent the number of cells recorded. Asterisks denote significance * 0.05, ** 0.01, *** 0.001 levels.

To study whether the pathogenic variants modify channel availability, the voltage dependence of inactivation was evaluated using 5-s pre-pulses depolarizations from − 100 to −20 mV preceding a 140-ms test potential to −10 mV (Figure 2A). No significant changes were observed in either the slope or shift in the mean-half inactivation potential (Figure 2B).

Altogether, these data indicate that M163T and R1182C mutations produce significant alterations in Cav2.3 channel gating, without changing the steady-state inactivation, which could result in reshaping Ca^2+^ influx along physiologically relevant membrane potentials that regulate neuronal firing and excitability.

### 2.5. The H151L Variant Exhibits a Decrease in Recovery from Inactivation

To determine the time constant of fractional recovery from inactivation, we used a two-pulse protocol separated by a varying interval ranging from 10 ms to 10 s and fitted with a single exponential function (Figure 2C). The H151L mutant had a significantly slower recovery from inactivation, with an increased time constant compared to Cav2.3 WT (4.04 ± 1.14 s vs. 0.78 ± 0.35 s). In contrast, no significant changes were observed for the M163T (0.27 ± 0.10 s) or R1182C (0.35 ± 0.11 s) variants. Consistent with this finding, H151L also showed a smaller fraction of channels recovered at several time points (ranging from 80 ms to 5 s), whereas the other pathogenic variants were not significantly different from Cav2.3 WT controls.

### 2.6. Structural Modeling of the Pathogenic Variants

**Figure 3:**
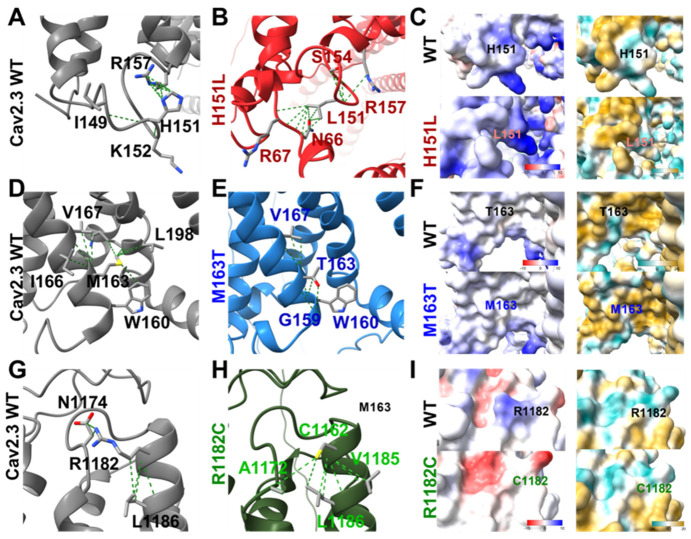
Modeling of Cav2.3 WT channels and H151L, M163T, and R1182C variants. (**A**) AlphaFold-predicted structure of Cav2.3 WT. (**B**) H151L variant. (**C**) Electrostatic surface potential (left panels) and molecular lipophilicity potential (right panels) for WT (upper) and H151L (lower). (**D**) Cav2.3 WT structure. (**E**) M163T variant. (**F**) Electrostatic surface potential (left) and molecular lipophilicity potential (right) for WT (upper) and M163T (lower). (**G**) Cav2.3 WT structure. (**H**) R1182C variant. (**I**) Electrostatic surface potential (left) and molecular lipophilicity potential (right) for WT (upper) and R1182C (lower). Electrostatic potential is displayed using a color scale ranging from −10 to 10 kcal mol^−1^ e^−1^ (red to blue). Molecular lipophilicity potential was mapped using a scale from −20 (hydrophilic, dark cyan) to +20 (lipophilic, dark gold). Electrostatic potential is displayed using a color scale ranging from −10 to +10 kcal mol^−1^ e^−1^ (red to blue). Molecular lipophilicity potential was mapped using a scale from −20 (hydrophilic, dark cyan) to +20 (lipophilic, dark gold).

Using AlphaFold prediction, we modeled the three missense variants (Figure 3). The three of them preserve the overall folding of the Cav2.3 channel, with moderate to high local confidence (predicted local distance difference test 70–90% for H151L and M163T; and >90% for R1182C) and a predicted template modeling score of 0.65 for each variant. The predictions suggest that none of the mutations are likely to induce major protein misfolding or destabilization. Compared to Cav2.3 WT, M163T introduces a polar threonine that forms a new hydrogen bond with the backbone carbonyl oxygen of a neighboring glycine residue, altering local surface hydrophilicity. H151L replaces histidine with a hydrophobic leucine, disrupting aromatic and polar interactions while increasing regional hydrophobicity. Finally, R1182C replaces a positively charged arginine with a neutral cysteine, reducing local positive electrostatic potential.

## 3. Discussion

De novo missense variants in *CACNA1E* give rise to broad clinical features ranging from severe, treatment-resistant developmental and epileptic encephalopathy to global developmental delay with intellectual disability, developmental regression, and autism spectrum disorder [13,14,15]. Interestingly, *CACNA1E* haploinsufficiency is poorly tolerated in the general population, with a pLI (probability of being loss-of-function intolerant) score of 1, suggesting that mutations are often pathogenic [23].

Most of the patients reported up to date have mutations clustered within the S6 segment of the different domains [13], producing Gain-of-Function (GoF) effects like hyperpolarizing shifts in their IV curves and increased current density [13,24,25].

In this report, we presented the functional characterization of three mutants, H151L, M163T, and R1182C, using electrophysiology which present Loss-of-Function (LoF) effects. To our knowledge, this is the first report linking Cav2.3 channelopathies to LoF mechanisms. M163T and R1182C present a depolarizing shift in their V_1/2_,act, reducing their availability at physiological potentials. In addition, R1182C also exhibits a decrease in peak current density, adding to its LoF.

For M163T, the AlphaFold model places this residue in proximity to the S4 segment of Domain I within the voltage-sensor domain. Although specific residue–residue interactions cannot be directly determined, substitution of methionine with a polar threonine may alter the local environment and contribute to altered voltage-dependent gating. Mutations in the voltage-sensor domain (S1–S4) of voltage-gated calcium (Cav) and other voltage-gated channels are well known to shift the energetic balance among channel conformations, thereby altering voltage dependence [26,27,28]. In this context, the depolarizing shift in V_1/2_,act observed for M163T is consistent with altered voltage-dependent gating, potentially reflecting stabilization of the resting state or destabilization of the activated state. This mechanism differs from that reported for GoF *CACNA1E* variants, which are instead associated with hyperpolarizing shifts in activation and increased current density [13].

In contrast, R1182C substitutes a positively charged residue, and the resulting loss of charge likely impairs voltage-dependent gating, consistent with the observed depolarizing shift in V_1/2_,act. Altogether, these findings indicate that the observed functional effects arise from alterations in channel gating, with no evidence of reduced membrane expression based on Gmax measurements.

Moreover, it is important to note that the severity and nature of the clinical outcome may depend on which biophysical property is primarily affected, rather than on overall LoF alone.

Studies in *CACNA1E* KO mice have demonstrated that loss of Cav2.3 function can alter neuronal excitability, synaptic plasticity, and even give resistance to certain seizure types while potentially facilitating others [29,30,31]. Cav2.3 channels are critical for generating dendritic plateau potentials and regulating burst firing in hippocampal and cortical neurons [32,33]. A LoF mutation like M163T, whose activation curve is shifted, could impair these plateau potentials, disrupting normal dendritic integration and activity-dependent synaptic plasticity, processes essential for learning and circuit refinement [34]. This may potentially underline the non-epileptic neurodevelopmental phenotype observed in the M163T patient.

In contrast to M163T and R1182C, the H151L variant does not alter voltage-dependent activation but instead significantly slows recovery from inactivation, resulting in a prolonged refractory period that is predicted to impair high-frequency firing and temporal summation of synaptic inputs. Structurally, substitution of histidine with a hydrophobic leucine eliminates potential polar and aromatic interactions by removing a side chain. These changes may influence local stability, affecting recovery from the inactivated state. Interestingly, Cav intracellular loops have been shown to contribute to inactivation gating [35,36].

This LoF is consistent with the patient’s neurological presentation, which includes adult-onset progressive cerebellar ataxia and spasticity, while the absence of epilepsy highlights the phenotypic variability associated with *CACNA1E* variants [13,14,15]. Impaired recovery kinetics may promote cumulative channel inactivation during sustained neuronal activity, thereby disrupting signal transmission in circuits that rely on tonic firing, including cerebellar Purkinje neurons and corticospinal pathways. Notably, H151L represents the first reported *CACNA1E* variant associated with absence of global developmental delay, further expanding the phenotypic spectrum of Cav2.3 channelopathies.

Finally, it is important to mention that further studies in native neuronal environments will be essential to elucidate the physiological and pathophysiological relevance of these alterations.

In summary, by using structural predictions with detailed electrophysiology and clinical data, we provide a mechanistic basis for 3 LoF pathogenic variants with phenotypic variability observed in *CACNA1E*-associated disorders. It is important to notice that the cellular and circuit basis of these clinical manifestations remains to be dissected to obtain further insights.

## 4. Materials and Methods

### 4.1. DNA Constructs

The human wild-type Cavα1E-3 subunit (GenBank: L29385.1) was kindly donated by Dr. Toni Schneider (University of Cologne, Cologne, Germany), and the rat Cavβ1b and Cavα2δ1 subunits were kindly provided by Dr. Terrance Snutch (University of British Columbia, BC, Canada). H151L, M163T, and R1182C variants were generated by site-directed mutagenesis performed by GenScript (https://www.genscript.com/, NJ, USA), and sequence fidelity was confirmed by sequencing of the whole coding region.

### 4.2. Cell Culture and Transfection

Human embryonic kidney tsA-201 cells were maintained at 37 C under a humidified atmosphere containing 5% CO_2_ and grown in Dulbecco’s modified Eagle’s medium (DMEM, Thermofisher, Waltham, MA, USA) containing 1% penicillin/streptomycin (Thermofisher, Waltham, MA, USA) and 10% fetal bovine serum (Thermofisher, Waltham, MA, USA). Cells were transiently transfected using the calcium phosphate method [37] with 3 µg of each plasmid encoding the following cDNAs: α1E-3-pcDNA3, H151L-pcDNA, M163T-pcDNA3, R1182C-pcDNA3, β_1b_-pcDNA3.1, and α_2_δ_1_-pcDNA3.1; and 0.5 µg of pEGFP to help identify transfected cells. The day after transfection, cells were washed and transferred to 30 C for 48–72 h before experiments.

### 4.3. Electrophysiology

Whole-cell voltage clamp recordings were performed at room temperature using an Axopatch 200B amplifier, a Digidata 1550B digitizer, and pCLAMP 11.2 acquisition software (Molecular Devices, San Jose, CA, USA). Currents were elicited by applying 400 ms pulses between −60 and +25 mV in 5 mV increments from a holding potential of −100 mV. Linear leakage and capacitive currents were subtracted online using a P/4 standard protocol. Current-voltage (I-V) relationships were generated from the peak current obtained during the pulses divided by the cell capacitance and were fitted with a modified Boltzmann equation: I  =  Gmax × (Vm  −  Vr)/(1  +  exp.(−(Vm  −  V_1/2_,act)/k)), where I is the peak current, Gmax is maximal conductance, Vm is the membrane voltage, V_1/2_,act is the half activation potential, Vr is the reversal potential, and *k* is the slope factor. The external recording solution contained (in mM): 2 CaCl_2_ (Sigma-Aldrich, Saint Louis, MO, USA), 137 CsCl (Thermofisher, Waltham, MA, USA), 1 MgCl_2_ (Sigma-Aldrich, Saint Louis, MO, USA), 10 HEPES (Sigma-Aldrich, MO, USA), 10 glucose (Sigma-Aldrich, Saint Louis, MO, USA). Solution was adjusted topH 7.4, with CsOH (Sigma-Aldrich, MO, USA. The pipette solution contained (in mM): 130 CsCl, 2.5 MgCl_2_, 10 HEPES, 10 EGTA (Sigma-Aldrich, Saint Louis, MO, USA), 3 ATP (Sigma-Aldrich, Saint Louis, MO, USA), 0.5 GTP (Sigma-Aldrich, Saint Louis, MO, USA). Solution was adjusted to pH 7.4, = with CsOH. Borosilicate glass pipettes (Sutter instruments, Novato, CA, USA) had a final tip resistance of 2–5 MΩ.

Steady-state inactivation curves were obtained by applying 5 s conditioning pulses from −100 to −20 mV in 5 mV increments, followed by a 140 ms test pulse to −10 mV. Curves were fitted with the equation I/I max  =  1/(1  +  exp(−(Vm  −  V_1/2_)/k)), where I/I max represents normalized current, Vm is the test membrane potential, V_1/2_ is the voltage for half inactivation, and *k* is the slope factor. Recovery from inactivation was determined using a two-pulse protocol. The first pulse (2 s) and second pulse (50 ms) were at 0 mV and separated by a varying interval ranging from 10 ms to 10 s. Traces were normalized to the maximum current during the first pulse for each sweep and plotted against time. Curves were fitted with a single exponential function.

### 4.4. Structural Modeling

A structural model of the Cav2.3 channel mutants was generated using the AlphaFold Protein Structure Prediction Server [38]. The amino acid substitution for each mutant was introduced directly into the human Cav2.3 WT input sequence (UniProt Q15878-1) prior to the structure prediction, and the predicted model was imported into UCSF ChimeraX [39] (version 1.10.1) for visualization and comparative analysis. The cryo-EM structure of the human Cav2.3 channel (RCSB PDB ID 8EPL) [40] was used as a template for alignment. The matchmaker command was used to align the AlphaFold predicted mutants and the human structure, which applies a sequence-guided structural superposition to minimize backbone deviations.

Local interactions within each mutated amino acid were examined using the contacts command with the true and reveal options enabled, allowing visualization of hydrogen bonds, van der Waals contacts, and steric interactions. Residues identified to interact with the mutated position were highlighted for further inspection.

To assess changes in the surface properties, electrostatic potential, and hydrophobicity maps were generated. Electrostatic surfaces were computed using the coulombic command to calculate the Coulombic electrostatic potential, while the molecular lipophilicity potential was visualized using the mlp command. These surfaces were displayed on the aligned structures to facilitate comparison between the mutant and wild-type models.

### 4.5. Statistics

Data are presented as means ± standard errors. Voltage errors were calculated, and cells with more than 5 mV in the IV curves were discarded. All data were analyzed for normality using D’Agostino and Pearson tests. Non-normally distributed data were analyzed via Kruskal–Wallis followed by Dunn’s multiple comparisons test. One-Way ANOVA comparisons. Significance was set at 0.05. Asterisks denote significance as follows: * *p*  <  0.05, ** *p * <  0.01, *** *p*  <  0.001.

## Data Availability

The data supporting the findings of this study are available within the article and could be made available from the corresponding author upon reasonable request.

## Abbreviations

The following abbreviations are used in this manuscript:

WT	Wild type
DEE	Epileptic encephalopathy
GDD	Global developmental delay
MRI	Magnetic resonance imaging
LoF	Loss-of-function
GoF	Gain-of-function
pLI	probability of being loss-of-function intolerant
